# Tissue nanotransfection and cellular reprogramming in regenerative medicine and antimicrobial dynamics

**DOI:** 10.3389/fbioe.2025.1558735

**Published:** 2025-06-18

**Authors:** Mohammed Youssef Shakra

**Affiliations:** Damietta Faculty of Medicine, Al-Azhar University, Damietta, Egypt

**Keywords:** tissue nanotransfection, cellular reprogramming, regenerative medicine, antimicrobial, wound healing

## Abstract

Tissue nanotransfection (TNT) is a novel, non-viral nanotechnology platform that enables *in vivo* gene delivery and direct cellular reprogramming through localized nanoelectroporation. This review synthesizes current advancements in TNT, outlining its device architecture, electroporation principles, and optimized delivery of genetic cargo, including plasmid DNA, mRNA, and CRISPR/Cas9 components. The mechanisms underlying TNT-mediated cellular reprogramming are critically evaluated, including transcriptional activation, epigenetic remodeling, and metabolic shifts, across three major reprogramming strategies—induced pluripotency, direct lineage conversion, and partial cellular rejuvenation. TNT demonstrates transformative therapeutic potential in diverse biomedical applications, including tissue regeneration, ischemia repair, wound healing, immunotherapy, and antimicrobial therapy. This review highlights TNT’s unique advantages over traditional gene delivery systems, namely, its high specificity, non-integrative approach, and minimal cytotoxicity, while also addressing existing limitations such as phenotypic stability and scalability. By integrating emerging data and identifying key translation challenges, this work positions TNT as a conceptual and technological advance in regenerative medicine and targeted gene therapy, offering a roadmap for future research and clinical implementation.

## 1 Introduction

Regenerative medicine has increasingly turned toward gene-based approaches to repair or replace damaged tissues. However, conventional gene delivery systems, particularly viral vectors, face substantial barriers, including immunogenicity, off-target effects, and limited *in vivo* applicability. Tissue nanotransfection (TNT) has emerged as a novel non-viral platform capable of delivering genetic material directly into tissues via localized nanoelectroporation, enabling cellular reprogramming *in situ* (Xuan et al., 2021).

Electroporation is a physical process that increases cell membrane permeability and the formation of transient membrane pores by applying an external electric pulse for milliseconds without affecting the cell viability. Electroporation-based techniques are used in numerous applications in medicine, food technology, and biotechnology (Batista Napotnik et al., 2024).

Cellular reprogramming is the conversion of a single lineage of somatic cells into another cell with a different identity using reprogramming factors, such as epigenetic modifications, transcription factors, metabolic factors, and non-coding RNAs. Direct reprogramming refers to cell fate conversion by electroporation without stem cell formation (Horisawa and Suzuki, 2020).

The broad therapeutic applications of TNT include tissue regeneration, ischemic repair, wound healing, immunomodulation, and antimicrobial therapy, exemplifying the broad translational potential of this technology. TNT exemplifies an interdisciplinary approach by integrating bioengineering, molecular biology, biotechnology, regenerative medicine, and immunology to enable *in vivo* cellular reprogramming and gene delivery (Alzate-Correa et al., 2022).

This review aims to synthesize the current landscape of TNT, exploring its device architecture, mechanisms of cellular reprogramming, therapeutic applications, and remaining translational challenges. By unifying these perspectives, the paper offers a roadmap for advancing TNT toward clinical implementation.

## 2 The structural components of a tissue nanotransfection (TNT) device

**FIGURE 1:**
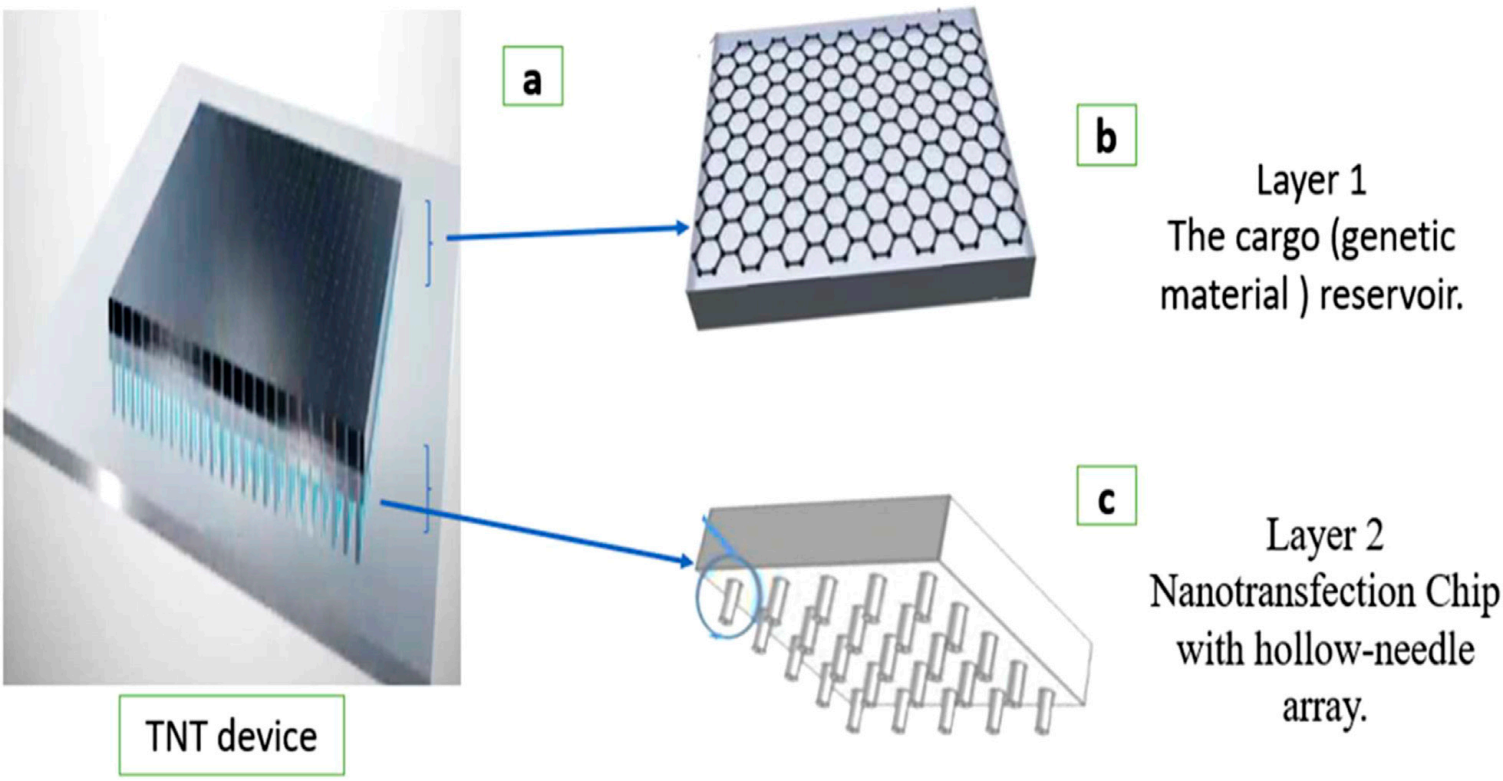
Diagram of the structural components of a tissue nanotransfection (TNT) device. **(a)** TNT device. **(b)** Layer 1: The cargo (genetic material) reservoir. The genetic material to be delivered is often stored in a reservoir. **(c)** Layer 2: The nanotransfection chip typically contains microneedles. Each needle has a central hollow channel through which genetic material can be transferred.

The tissue nanotransfection (TNT) device consists of a hollow-needle silicon chip mounted beneath a cargo reservoir containing a genetic material, for example, a plasmid solution. This device is placed directly on the skin or target tissue (Figure 1). The cargo reservoir is connected to the negative terminal of an external pulse generator, while a dermal electrode connected to the tissue serves as the positive terminal (Xuan et al., 2021; Lemmerman et al., 2021).

When electrical pulses are applied, the hollow needles concentrate the electric field at their tips, temporarily porating nearby cell membranes and enabling the targeted delivery of the charged genetic material into the tissue. This configuration allows precise, localized, non-viral, and efficient *in vivo* gene delivery (Xuan et al., 2024).

The optimization of electrical pulse parameters—such as voltage amplitude, pulse duration, and inter-pulse intervals—is critical for maximizing delivery efficiency while preserving cellular viability during the nanotransfection process (Huang et al., 2019).

The sterilization of TNT devices is an essential step to ensure their safety for use in biological and medical applications. Among the most frequently applied sterilization processes in medical devices are ethylene oxide gas sterilization and gamma irradiation. Ethylene oxide sterilization preserves the interior architecture of the nanodevices (Hyder et al., 2020).

## 3 Electroporation-based delivery system

There are three delivery systems for cell reprogramming: biological, chemical, and physical. The delivery system should be safe and effective to achieve successful reprogramming with specific reprogramming factors (Umeyama et al., 2024).

Biological delivery systems frequently rely on genetically engineered viruses due to their high transduction efficiency and ability to mediate stable gene expression. However, these viral vectors present certain challenges referred to as “off-target” effects, such as immunotoxicity and unintended gene expression in non-target tissues, which remain significant barriers to safe and effective clinical application (Li et al., 2023).

Chemical (non-viral) gene delivery systems offer several advantages, such as ease of production, the ability to accommodate large genetic payloads, and reduced immunogenicity compared to viral vectors. However, their clinical application remains limited due to several critical challenges. These include low transfection efficiency *in vivo* due to poor cellular uptake, inefficient endosomal escape, poor targeting specificity, instability in physiological environments, and cytotoxicity associated with commonly used polymers (Piperno et al., 2021).

Physical delivery systems such as electroporation and mechanical disruption act by membrane disruption mechanisms. Nanoelectroporation is an efficient and fast transfection method that does not affect cell viability (Wang et al., 2022).

DNA probes, small interfering RNAs (siRNAs), and plasmids are negatively charged and cannot pass directly through the membranes of different cells because they have the same charge. To deliver these molecules without irreversible damage, *in vitro* electroporation-based systems have been developed, such as nanostraw-based devices, nanochannel-based devices, and flow-through microfluidic chips (Hur and Chung, 2021).

Electroporation is a physical mechanism by which an external electric field promotes cell membrane permeability. The electric field produces thermal fluctuations that rearrange the molecules in the phospholipid bilayer and form hydrophilic pores to allow molecules and ions to cross in both directions. The pores typically reseal, leaving the cell membrane normally intact after the removal of the electrical pulse (Li et al., 2022).

TNT employs a highly localized and transient electroporation stimulus through nanochannel interfaces that are designed to create reversible nanopores in the plasma membrane. These nanopores typically reseal within milliseconds or a few seconds, depending on cell type and membrane characteristics. The short duration of pore opening limits the opportunity for cell damage and cytotoxicity (Xuan et al., 2021).

## 4 Plasmid DNA, mRNA, and CRISPR/Cas9 in transfection

The genetic material selected for transfection should be prepared, purified, and optimized for delivery. Current research prioritizes plasmid DNA and mRNA for TNT applications due to their transient expression profiles, which minimize genomic integration risks like permanent alterations to the genome (Moradian et al., 2020; Stewart et al., 2018).

Plasmid DNA is a vector for transfection. DNA plasmids containing recombinant genes and regulatory elements can be transfected into cells to study gene function, regulation, and effects of gene expression on the health and life cycle of cells. DNA plasmid transfection requires nuclear entry before gene expression. Highly supercoiled, circular DNA plasmids are more efficient than linear DNA plasmids for performing transient transfection because circular plasmids are not vulnerable to exonucleases, while linear DNA fragments are quickly degraded by these enzymes (Oliynyk and Church, 2022).

Messenger RNA (mRNA) transfection is a versatile technique that can be performed both *in vitro* and *in vivo* using cationic lipid-mediated delivery or electroporation. mRNA transfection allows for direct protein translation in the cytoplasm without requiring nuclear entry, making it simpler, faster, and more efficient than DNA plasmid transfection (Qin et al., 2022).

Synthetic transcriptional control has emerged as a transformative approach for reprogramming gene expression *in vivo*. The advent of CRISPR/Cas9-based technologies, particularly catalytically inactive dCas9 fused to transcriptional or epigenetic effector domains, has revolutionized the field by offering a more programmable, modular, and multiplexable platform for endogenous gene regulation (Pandelakis et al., 2020).

Synthetic transcription factors guided by RNA sequences represent a transformative tool in gene regulation and synthetic biology. These engineered proteins are designed to modulate gene expression with high specificity and tunability, offering a wide range of applications in research, biotechnology, and medicine. They induce epigenomic modifications or transcriptional changes at precise loci, with potential applications in treating complex diseases by restoring disrupted gene regulatory networks (Bhatt et al., 2024).

## 5 Types of cellular reprogramming

Cellular reprogramming encompasses various approaches, including induced pluripotent stem cells (iPSCs), direct reprogramming (transdifferentiation), and partial reprogramming (cellular rejuvenation) (Su et al., 2023). iPSC reprogramming involves transforming somatic cells into a pluripotent state using transcription factors. The produced stem cells may be associated with a risk of immunogenicity and tumorigenicity, epigenetic and genetic abnormalities, low conversion, and unstable differentiation (Qiao et al., 2020).

Direct reprogramming, also referred to as transdifferentiation, involves the conversion of one somatic cell type into another without passage through a pluripotent state, offering a more direct, rapid, and potentially safer strategy for cell replacement therapies and regenerative medicine without inducing uncontrolled proliferation or dedifferentiation (Wang et al., 2021).

*In vivo*, the overexpression of genetic factors can stimulate cell lineages to repair damaged tissue without tumorigenesis, risk of contamination, or cell transplantation. This direct lineage conversion technology holds great practical promise (Han et al., 2022).

Partial reprogramming through transient OSKM activation [octamer-binding transcription factor (Oct4), sex-determining region Y-box 2(Sox2), Krüppel-like factor 4 (klf-4), cellular Myc myelocytomatosis (c-Myc)] has demonstrated the ability to reverse aging-related changes in senescent cells for treating age-related diseases without altering cell identity (Cipriano et al., 2024).

This approach resets epigenetic markers like DNA methylation clocks, reduces aging-associated transcriptional dysregulation, and restores serum metabolites to youthful levels. Additionally, mitochondrial rejuvenation via chemical cocktails enhances oxidative phosphorylation and mitochondrial function in aged fibroblasts (Chondronasiou et al., 2022).

Telomeres are the protective caps at the ends of chromosomes that shorten with age and cellular division. Telomerase activation and telomere lengthening are key outcomes of partial cellular reprogramming. This is achieved through transient expression of reprogramming factors (such as OSKM). This phenomenon involves epigenetic modifications that create a more open chromatin state at telomeres, facilitating telomere elongation. As a result, cells exhibit improved genomic stability and reduced markers of cellular aging, highlighting the potential of partial reprogramming for safe, targeted rejuvenation (Rubtsova et al., 2024).

## 6 Mechanism of cellular reprogramming

The molecular mechanisms underlying cellular reprogramming involve complex changes in how genes are expressed and how cells function. Key factors for distinct types of cellular reprogramming include transcription factors that activate new genes while suppressing old ones, changes in chromatin structure, and epigenetic modifications that influence gene activity. DNA methylation patterns are also altered to stabilize these changes. Additionally, non-coding RNAs help fine-tune gene expression, and metabolic shifts support the transformation process. All these mechanisms work together to convert one cell type into another (Peng et al., 2023).

The lineage-specific transcription factors, particularly pioneer factors, initiate reprogramming by binding to closed chromatin regions and enabling access to additional transcriptional regulators. These factors work synergistically to activate target gene networks while repressing original cell identity programs. Chromatin accessibility is rapidly and dynamically altered upon reprogramming induction, with changes occurring at distal regulatory elements and enhancer regions (Hashimoto et al., 2019).

During cellular reprogramming, activating histone marks such as H3K4me3 (trimethylation at lysine 4 of histone H3) and H3K27ac (acetylation at lysine 27 of histone H3) are deposited at promoters and enhancers of target lineage-specific genes, promoting open chromatin and active transcription. In contrast, repressive marks like H3K27me3 (trimethylation at lysine 27) and H3K9me3 (trimethylation at lysine 9) are removed from these newly activated regions and redistributed to genes associated with the original cell identity. This coordinated remodeling of histone modifications plays a crucial role in silencing the previous cell program while activating the new one, enabling the epigenetic reprogramming required for lineage conversion or rejuvenation (Bae et al., 2025).

During cellular reprogramming, DNA methylation patterns are extensively remodeled, with the demethylation of lineage-specific genes. This reconfiguration is essential for stable transcriptional reprogramming and involves enzymes such as Dnmt3a (DNA methyltransferase 3a), non-coding RNAs, including microRNAs [miRNAs like miR-133, miR-9/9*, and miR-124, as well as long non-coding RNAs such as lnc-NR2F1 (nuclear receptor subfamily 2 group F member 1)] to fine-tune gene expression post-transcriptionally. The non-coding RNAs modulate chromatin states, enhancing the efficiency and specificity of the reprogramming process by regulating transcription and chromatin dynamics (Horisawa et al., 2023; Hunkler et al., 2022).

Metabolic reprogramming during somatic cell reprogramming is a pivotal mechanism. In the early stage of reprogramming, a transient increase in the oxidative phosphorylation (OXPHOS) rate is an essential event. This OXPHOS burst then declines, which reduces reactive oxygen species (ROS) production, followed by maintaining glycolytic metabolism in the later phase of reprogramming (Ishida et al., 2020).

Through the reprogramming technique, reprogrammed cells acquire the functional characteristics of target cells and exhibit their biological properties, for example, the ability to secrete specific proteins, form appropriate cellular structures, or conduct specialized functions. The reprogrammed cells contribute to tissue regeneration and repair processes by integrating into the existing tissue and replacing damaged or lost cells (Grath and Dai, 2019).

Reprogramming factors exert their effects predominantly in specific target cells due to the presence of a permissive molecular and epigenetic environment that facilitates their function. These target cells often exhibit accessible chromatin landscapes at key lineage-specific genes, enabling the binding and transcriptional activation by introduced factors. In contrast, non-target cells typically harbor restrictive epigenetic modifications—such as DNA methylation and repressive histone marks—that inhibit factor binding or activity (Zhu and Nie, 2025).

Moreover, the transcriptional networks and cofactors endogenous to target cells can synergize with reprogramming factors to promote cell fate transitions, whereas such supportive elements are often absent in other cell types. Cellular plasticity and developmental stage further influence susceptibility to reprogramming, with progenitor-like or more plastic cells showing greater responsiveness. Collectively, these intrinsic differences ensure that reprogramming factors selectively induce changes in target cells while limiting unintended effects in non-target populations (Kuang et al., 2022).

Studies on the behavior of transfected cells over time show distinct outcomes depending on the cell type, cell cycle stage, transfection method (chemical, electroporation, and viral), cell health, and purpose (Liu et al., 2023).

Transient transfection leads to short-term gene expression, typically lasting from a few hours to a few days. The introduced genetic material exists in the cell only for a limited period, remains episomal, and is not integrated into the genome. It is not passed from generation to generation and is diluted or degraded by nucleases during cell division. This approach is useful for rapid functional studies or protein production, but it does not sustain long-term effects (Mousavi Kahaki et al., 2024).

Stable transfection achieves long-term gene expression by integrating the transgene into the host genome, allowing sustained expression in the transfected cell and its progeny. The persistent expression of introduced exogenous DNA through multiple generations can be useful for the production of recombinant proteins or therapeutic applications requiring persistent gene activity (Chong et al., 2021).

Pancreatic acinar cells directly reprogrammed to induced β-cells undergo rapid epigenetic and transcriptional changes within the first 10 days, followed by continued maturation over 1–2 months marked by activation of β-cell-specific genes like urocortin 3. After this phase, they maintain a stable β-cell identity and function for at least 7 months, showing strong expression of β-cell markers, loss of acinar traits, and integration into islet structures, closely resembling native β-cells in both gene expression and function (Li et al., 2014).

Confirmation of successful cellular reprogramming involves a combination of molecular, epigenetic, functional, and morphological assessments. Gene expression analysis (via RT-qPCR or RNA-seq) verifies the upregulation of target cell-specific genes and the downregulation of original cell markers (Huang et al., 2021), while immunostaining and Western blotting confirm the presence of key lineage-specific proteins (Sun et al., 2022). Epigenetic changes are evaluated through chromatin immunoprecipitation (ChIP) to assess histone modifications and DNA methylation analysis to confirm remodeling consistent with the new cell identity (Liu et al., 2025). Functional assays, such as differentiation potential, electrophysiological studies, or metabolic activity, validate the capabilities of the reprogrammed cells (Li et al., 2025). Flow cytometry is used to detect surface markers, and morphological evaluation under microscopy helps identify changes in cell shape that are typical of the target lineage (Huang et al., 2025). In some cases, single-cell RNA sequencing is employed to assess transcriptional identity and population uniformity at the single-cell level (Van de Sande et al., 2023).

## 7 Applications for tissue nanotransfection, reprogramming, and electroporation

**FIGURE 2:**
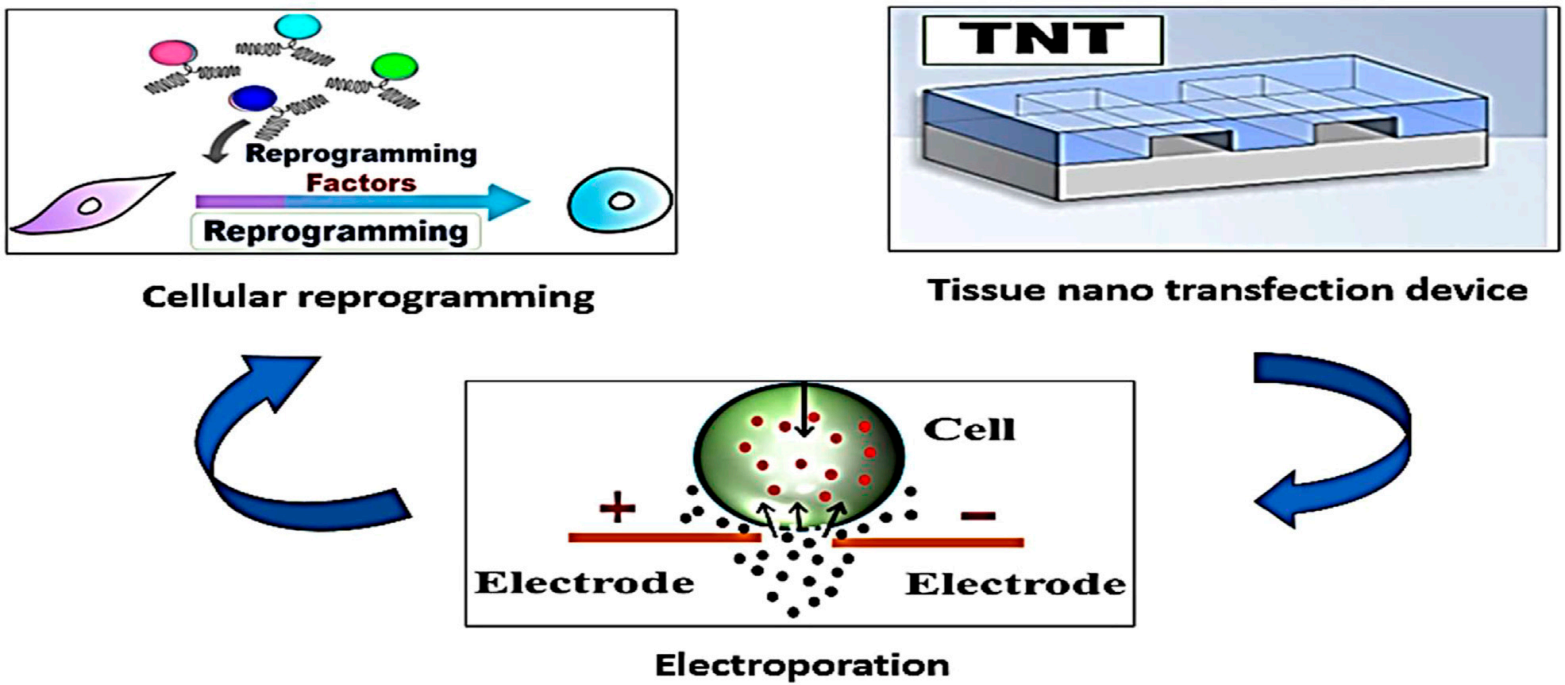
The tissue nanotransfection technique produces cell membrane electroporation and introduces reprograming factors into diverse types of somatic cells to change their identity and phenotype. This technique has several medical applications, such as tissue regeneration and wound healing.

TNT can deliver therapeutic genes directly into affected cells without the need for sophisticated laboratory equipment. This approach has potential applications in cardiovascular diseases, neurodegenerative diseases, and genetic disorders (Figure 2). Tissue nanotransfection by reprogramming cells can modulate cellular functions, correct genetic mutations, or stimulate the production of therapeutic proteins (Liu et al., 2024).

### 7.1 Tissue regeneration

In a murine wound model, TNT was used to specifically deliver the formyl peptide receptor 1 (FPR1) open reading frame (ORF) to myeloid cells localized at the wound edge. This targeted delivery reactivated the annexin A1–FPR1 signaling axis, a pathway typically dormant in adult tissues but active in fetal skin. TNT induced a pro-regenerative phenotype in myeloid cells, characterized by enhanced anti-inflammatory responses and improved extracellular matrix (ECM) organization. These changes collectively resulted in accelerated wound closure with significantly reduced fibrosis and scarring (Srivastava et al., 2023).

TNT has been utilized for muscle regeneration after volumetric muscle loss. The muscle was surgically exposed, and the TNT nanochip was placed directly on the muscle surface. TNT was used to deliver MyoD, a master myogenic transcription factor, directly into the injured muscle tissue, promoting the differentiation of local cells into muscle lineage. TNT enables the reprogramming of resident non-muscle cells, such as fibroblasts within fibrotic scar tissue, into functional myogenic cells, thereby supporting the formation of new muscle fibers within the damaged region. TNT significantly enhances muscle recovery, as demonstrated by improved maximal dorsiflexion torque, greater resistance to fatigue, hypertrophy, increased muscle mass, and improved contractile force (Clark et al., 2022).

### 7.2 Ischemia

In murine models of injury-induced ischemia, tissue nanotransfection rescued ischemic tissue by delivering reprogramming factors EFF [E-26 transformation-specific variant 2 (Etv2), friend leukemia integration 1 (Fli1) and forkhead box C2 (Foxc2)] directly into skin cells. TNT targets dermal fibroblasts, epidermal keratinocytes, and follicular cells for reprogramming with electrical stimulation (ten pulses of 250 V for 10 milliseconds per pulse). The induced endothelial cells (iECs) from reprogramming promote neovascularization in the ischemic area. This increased vascular density enhances blood flow, counteracts tissue necrosis, and supports the functional reperfusion of injured tissue, demonstrating its strong therapeutic potential in ischemic disease (Gallego-Perez et al., 2017).

TNT is used in the treatment of ischemic stroke to deliver a cocktail of genes (Etv2, Foxc2, and Fli1) to fibroblasts through injection into the subarachnoid space above the stroke-affected sensorimotor cortex, reprogramming the fibroblasts into induced endothelial cells (iECs). This reprogramming is achieved using a pulsed electric field of approximately 27.5 V/mm, with a pulse duration of 35 milliseconds, applied in ten pulses. Intracranial delivery of the induced endothelial cells (iECs) increases perfusion in both healthy and stroke-affected brains, reduces infarct volume by approximately 70%, and improves motor recovery by up to 90% in mice (Lemmerman et al., 2021).

### 7.3 Wound healing

In diabetic wounds, chronic hyperglycemia leads to epigenetic dysregulation, including hypermethylation of the phospholipase C gamma 2 gene (PLCγ2) promoter, which suppresses PLCγ2 expression and impairs angiogenic responses. This epigenetic silencing diminishes the efficacy of vascular endothelial growth factor (VEGF)-based therapies. TNT delivers a CRISPR-dCas9-based demethylation cocktail to the ischemic wound edge. This approach reactivates PLCγ2 expression by promoter demethylation, restoring angiogenic signaling via activation of the p44/p42 mitogen-activated protein kinase to the hypoxia-inducible factor 1-alpha signaling pathway. As a result, TNT enhances neovascularization, accelerates wound closure, and rescues impaired healing in diabetic ischemic wounds (Verma et al., 2025).

Long-term hypoxia and infection in chronic wound tissue lead to epigenetic gene silencing in the microenvironment due to DNA hypermethylation in the edge tissue of chronic wounds. Topical administration of the hypomethylating agent 5-azacytidine by using a CRISPR/dCas9-based approach for tissue nanotransfection improves wound healing and demethylates genes in ischemic wounds (Singh et al., 2022).

### 7.4 Immunotherapy

Tissue nanotransfection facilitates the *in situ* generation and delivery of engineered extracellular vesicles functionalized with intercellular adhesion molecule-1 (ICAM-1) ligands. These ligands specifically bind to CD11b/CD18 receptors expressed on myeloid-derived suppressor cells and tumor-associated macrophages, ensuring the selective delivery of these cells within the immunosuppressive microenvironment of the tumor. These EVs are loaded with microRNA 146a (miR-146a) and glucose transporter 1 (Glut1) transcripts. miR-146a acts by suppressing anti-inflammatory signaling pathways by the inhibition of interleukin-1 receptor-associated kinase 1 (IRAK1) and tumor necrosis factor receptor-associated factor 6. Glut1 mRNA promotes metabolic reprogramming by enhancing glucose uptake and glycolysis. This approach aims to drive intratumoral repolarization of myeloid cells toward a pro-inflammatory state, which is complemented by increased T-cell infiltration and reduced tumor size and metastatic burden (Duarte-Sanmiguel et al., 2023).

In an experimental murine study, TNT delivered anti-miR-126 oligonucleotides directly into tumors, silencing miR-126 and disrupting its transfer via extracellular vehicles (EVs) to tumor-associated macrophages (TAMs). This reprogrammed TAMs from a pro-tumoral (M2-like) to an anti-tumoral state, leading to complete tumor regression and prolonged survival in mice. TNT offers a potent, localized gene therapy approach for remodeling the tumor immune microenvironment and improving cancer treatment outcomes (Gordillo et al., 2023).

### 7.5 Neuropathies

Topical cutaneous intracellular transport of achaete-scute complex-like 1 (Ascl1), brain-specific homeobox/POU domain protein 2 (Brn2), and myelin transcription factor 1 (Myt1) genetic molecules by TNT succeeded in the direct conversion of skin fibroblasts into activated neuronal cells *in vivo* and initiated skin stroma neurotrophic enrichment by rescuing preexisting nerve fibers in chronic diabetic wounds. TNT resulted in the elevation of endogenous nerve growth factor and neurotrophin-3 (Nt3) due to the elevated expression of the protein-encoding gene product (PGP9.5+) in mature nerve fibers (Roy et al., 2020).

Ascl1 promotes histone acetylation, ensuring chromatin remains in an open, transcriptionally active state. In coordination with Brn2 and Myt1l, Ascl1 drives the commitment to a neuronal lineage while repressing the expression of non-neuronal genes. Furthermore, Ascl1 regulates the expression of genes essential for neuronal differentiation, including those involved in synaptogenesis, ion channel formation, and neurotransmitter production (Păun et al., 2023).

TNT successfully facilitated voltage-dependent delivery of plasmid DNA to the sciatic nerve in mice without impairing either behavioral or electrophysiological function, as evidenced by preserved toe-spread reflex, pinprick response, and nerve conduction parameters. TNT enabled targeted delivery of vasculogenic reprogramming factors—Etv2, Foxc2, and Fli1 (EFF)—to sites of sciatic nerve injury within the epineurium, the nerve’s protective outer layer, allowing precise modulation of transfection resulting in enhanced vascularization, reduced macrophage infiltration, and accelerated electrophysiological recovery compared to controls (Moore et al., 2020).

### 7.6 Lymphedema

In a mouse model of lymphedema, TNT was used prophylactically to deliver prospero homeobox 1 (Prox1), a master regulator of lymphangiogenesis, to the site of lymphatic injury. This focal approach significantly reduced tail swelling, improved lymphatic clearance, increased lymphatic vessel density, and decreased both inflammation and fibrosis compared to controls. TNT-delivered Prox1 outperformed vascular endothelial growth factor C (VEGF-C) therapy, owing to its upstream regulatory role and its localized action. TNT, especially in surgical settings, presents it as a promising strategy for preventing lymphedema and improving lymphatic repair (Mohan et al., 2024).

TNT plays a significant role in therapeutic intervention in lymphedema. The technique uses a TNT2.0 silicone chip to deliver plasmid DNA directly into the skin of the mouse tail using a brief, focused square wave electric pulse (ten electrical pulses, each lasting 10 milliseconds, at 250 V). The fluorescein amidite (FAM)-labeled DNA was employed as a tracer to track and confirm the successful delivery of genetic material in the mouse tail lymphedema model. The outcomes of local cutaneous gene delivery included improvements in lymphatic functions and reductions in the volume of the tail of the mouse, as observed by near-infrared laser lymphangiography and real-time lymphatic flow without immunogenic or oncogenic risks (Hassanein et al., 2021).

### 7.7 Cosmetic medicine

The mechanism of dermal photoaging involves the destruction of collagen and extracellular matrix (ECM) proteins and wrinkle formation. Extracellular vesicles formed from human skin fibroblasts loaded with mRNA for α1 type-I collagen in the extracellular matrix were used to generate collagen-protein grafts by cellular nanotransfection. The graft succeeded in reducing wrinkle formation by prolonged and uniform synthesis of collagen, which replaced the depleted collagen (You et al., 2023).

### 7.8 Antimicrobial

TNT is a non-viral gene delivery platform that effectively treated *Staphylococcus aureus* biofilm-associated wound infections by delivering the cathelicidin antimicrobial peptide (CAMP) gene encoding the antimicrobial peptide LL-37 directly into infected wound in a mouse model. TNT enabled localized gene expression, significantly reducing bacterial biofilms and promoting antimicrobial activity. TNT also enhanced immune response and wound healing by increasing macrophage recruitment, angiogenesis, and anti-inflammatory signaling. This study highlights TNT’s potential as a multifunctional, non-antibiotic gene therapy for combating resistant infections while supporting tissue repair (Cuellar-Gaviria et al., 2024).

An electroporation-based membrane coating strategy was used for osteomyelitis therapy. Nanoparticles (NPs) with osteoconductive tricalcium phosphate Ca3(PO4) and bactericidal titanium oxide (TiO2) were phagocytosed by macrophages and then exposed to an electric field for electroporation to obtain macrophage membrane-coated NPs. These nanoparticles adsorbed bacteriotoxin, regulated inflammatory cytokines, and exerted anti-inflammatory and antibacterial effects. The excellent bactericidal activity of macrophage membrane-coated NPs was attributed to the macrophage membrane and the reactive oxygen species (ROS) produced by TiO2, which were effective against *Escherichia coli* (*E. coli*), *Staphylococcus aureus*, and methicillin-resistant *Staphylococcus aureus* (MRSA). An electroporation-based membrane coating strategy can be used with diverse types of nanoparticles and cells (Shi et al., 2021).

CRISPR/Cas systems offer a versatile platform for next-generation antiviral therapies. Their ability to be programmed to target and cleave specific viral DNA or RNA sequences enables direct inhibition or elimination of a wide range of viruses, including acute RNA viruses like SARS-CoV-2 and influenza, chronic viruses such as hepatitis B, and latent viruses like HIV-1. The feasibility of using various Cas effectors (Cas9, Cas12, and Cas13) in both *in vitro* and *in vivo* settings enables suppression of viral replication. The use of TNT enhances *in vivo* delivery of CRISPR components.Cas13 acts against RNA viruses by using a programmable CRISPR RNA (crRNA) to specifically bind and cleave single-stranded viral RNA inside infected cells. It supports multiplex targeting to minimize viral escape and operates independently of host machinery, offering high specificity and low off-target effects (Baddeley and Isalan, 2021).

Microbial biofilms pose a major challenge in treating bacterial and fungal infections due to their high resistance to conventional antimicrobial therapies. The integration of antimicrobial photodynamic therapy with pulsed electric fields (PEFs) has further enhanced treatment efficacy. PEFs are a more advanced, specialized form of electroporation that can disrupt microbial membranes, promote the formation of reactive species, alternate the intracellular calcium ion (Ca2+) concentration, and reduce protein leakage and apoptosis, contributing to a broader antimicrobial effect (Martins Antunes de Melo et al., 2021).

*In vitro*, PEFs significantly reduce the viability of *Candida* albicans. When PEFs were combined with antifungal drugs such as amphotericin B or naftifine, over 90% of *C. albicans* colony-forming units were eliminated in a single procedure, indicating a rapid and synergistic effect. Additionally, PEF treatment caused notable morphological changes in the fungal cells, including aggregation and a reduction in average cell size by up to 53%. These findings suggest that PEFs, especially in combination with antifungal agents, offer a promising and potentially less toxic alternative or adjunct to conventional antifungal therapies (Novickij et al., 2015).

An *in vitro* study investigated the combined effects of silver nanoparticles (SNPs) and electroporation (4 pulses at 1 Hz, 700 V/cm, 100 ms duration) on *Leishmania major*, using promastigotes and infected macrophages. Electroporation enhances the antileishmanial properties of SNPs, potentially improving their effectiveness by increasing the entry of silver particles into promastigotes and amastigotes without harming macrophages. Electric pulses promote the release of silver cations, which results in the distortion of promastigote shape and internal organelles, reduction of metabolic activity and viability, and reduces the amastigote infection index (Dolat et al., 2015).

DNA vaccine encoding the *Toxoplasma gondii* surface antigen-related sequence 13 (SRS13) protein elicits strong immunogenic responses and offers significant protection against chronic toxoplasmosis in BALB/c mice. Immunization via both intramuscular injection and intradermal electroporation induced robust anti-SRS13 IgG responses and elevated IFN-γ production, particularly in CD8^+^ T cells. Notably, the intradermal route combined with electroporation resulted in a stronger cellular immune response and greater reduction in brain cyst burden than intramuscular delivery. These findings suggest that the SRS13-based DNA vaccine is a promising candidate for further development as a protective strategy against toxoplasmosis (Gül et al., 2024).

Delivery of a combination of the circumsporozoite protein of *Plasmodium falciparum* (PfCSP) and *Plasmodium falciparum* surface antigen 25 (Pfs25) into mice by electroporation successfully induced effective immune responses against both Pfs25 and PfCSP. The specific antibodies induced against the combination of PfCSP and Pfs25 DNA have a protective effect against infection and reduce mosquito transmission (Cao et al., 2022).

## 8 Tissue nanotransfection challenges

Despite its substantial promise, TNT faces several critical challenges that must be addressed to enable widespread clinical translation. A primary limitation lies in the need for cell-type-specific reprogramming protocols, as the transcriptional and epigenetic landscapes vary significantly across tissue contexts, influencing reprogramming efficiency and fidelity (van Gurp et al., 2022).

Ensuring long-term stability, phenotypic maintenance, and functional integration of reprogrammed cells remains a major concern, particularly in dynamic *in vivo* environments where microenvironmental cues and immune responses may alter cellular behavior. Additionally, optimization of electroporation parameters, such as voltage, pulse duration, and frequency, is essential to balance transfection efficiency with cell viability and minimize off-target effects (Almeida et al., 2025).

Manufacturing and sterilization of TNT devices under good manufacturing practice (GMP) conditions, along with standardization of genetic cargo preparation, are necessary to meet regulatory standards. Finally, comprehensive preclinical studies are needed to ensure their safety and efficacy in human applications (Yadav et al., 2024).

## 9 Conclusion

Tissue nanotransfection (TNT) is a non-viral gene delivery and *in vivo* cellular reprogramming technology. By integrating nanoengineered electroporation platforms with diverse genetic cargos, TNT enables direct, localized, and efficient modulation of cell fate. This review has elucidated the mechanistic foundations, device architecture, and molecular reprogramming strategies underpinning TNT, as well as its demonstrated therapeutic utility across a broad spectrum of biomedical applications, including tissue regeneration, vascular repair, wound healing, immunomodulation, and antimicrobial interventions. Continued interdisciplinary efforts across bioengineering, regenerative medicine, and molecular biology will be essential to refine TNT platforms and realize their clinical applicability.

## Glossary

TNT: Tissue nanotransfection
DNA: Deoxyribonucleic acid
RNA: Ribonucleic acid
siRNAs: small interfering RNAs.
miRNA: microRNA
mRNA: Messenger RNA
OSKM: Oct4, Sox2, Klf4, c-Myc
klf-4: Krüppel-like factor four
CRISPR: Clustered Regularly Interspaced Short Palindromic Repeats
Cas9: CRISPR-associated protein nine
Oct4: Octamer-binding transcription factor
c-Myc: Cellular Myc Myelocytomatosis
Sox2: Sex-determining Region Y-Box 2
H3K4me3: trimethylation at lysine four of histone H3
H3K27ac: acetylation at lysine twenty-seven of histone H3
H3K27me3: trimethylation at lysine twenty-seven
H3K9me3: trimethylation at lysine nine
lnc-NR2F1: Long non-coding RNA-Nuclear Receptor Subfamily 2 Group F Member 1
OxPhos: oxidative phosphorylation
ROS: reactive oxygen species
PLCγ2: phospholipase C gamma two gene
FPR1: formyl peptide receptor one
Fli1: Friend leukemia integration one
Foxc2: Forkhead box C2
Etv2: E-twenty-six Transformation-Specific Variant 2
Ascl1: Achaete-Scute Complex-like one
Brn2: Brain-specific homeobox/POU domain protein two
Myt1: Myelin transcription factor one
TAMs: tumor-associated macrophages
M2: Macrophages 2
miR-146a: MicroRNA-146a
ChIP: chromatin immunoprecipitation.
Glut1: Glucose transporter one
Nt3: Neurotrophin-3
PGP9.5: Protein-encoding gene product 9.5
Prox1: Prospero homeobox one
FAM: fluorescein amidite
NPs: Nanoparticles
Ca3(PO4): tricalcium phosphate
TiO2: titanium oxide
PfCSP: circumsporozoite protein of Plasmodium falciparum
Pfs25: Plasmodium falciparum surface antigen twenty-five
PEFs: nanosecond pulsed electric fields.
